# The Progestin Revolution: progestins are arising as the dominant players in the tight interlink between contraceptives and bleeding control

**DOI:** 10.1186/s40834-020-00142-5

**Published:** 2021-01-07

**Authors:** Donna Shoupe

**Affiliations:** Keck School of Medicine, University of Southern California, Los Angeles, California USA

## Abstract

Since the introduction of the first modern contraceptive methods, the interlink between bleeding control and contraceptive development has been a dominant and critical factor. This interplay has led to the development of safer and better contraceptive methods that are often used to control bleeding in both women with normal bleeding patterns as well as in those suffering from heavy menstrual bleeding (HMB). The success of progestin-only methods, such as hormonal IUDs or progestin dominant oral contraceptives in substantially decreasing and controlling menstrual bleeding, has led to development of multiple progestin-only protocols for the sole purpose of bleeding control. These include protocols designed to stop acute heavy bleeding as well as manage long-term bleeding. Recent publications describe a variety of protocols using high dose oral progestin pills with or without a medroxyprogesterone acetate (MPA) injection that demonstrate high effectiveness and good tolerability. Comparted to many other progestins, MPA is not converted in part into ethinyl estradiol and appears to have a progestin-only advantage. Norethindrone acetate (NET acetate) is converted in part to ethinyl estradiol and therefore is an especially good option for bleeding control in patients with low estrogen levels that would benefit from estrogen replacement (such as in premenopausal women with premature menopause or hypothalamic hypogonadism).

## A brief history of the tight interlink between products designed for bleeding control and contraceptives

The first two FDA approved contraceptive methods used in this country were originally medications approved for non-contraceptive indications. The Enovid pill received regulatory approved in 1957 as a treatment for menstrual disorders and infertility [1] and was marketed in the US to treat “disturbances of menstruation”. Depo-medroxyprogesterone acetate was originally approved for treatment of endometrial and renal cancer [2] but it was widely used for bleeding control for more than 30 years before it was approved for contraception by the FDA in 1992. While Enovid contained dangerously high levels of a progestin [9.85 mg norethynodrel] and an estrogen [150 μg mestranol], it luckily had success in the market. Amid storms of controversies, the US FDA finally approved Enovid as the US’s first oral contraceptive pill in June 1960. After the 1965 Supreme Court Decision, Griswold v. Connecticut that revoked state laws preventing the distribution of contraceptives, the oral contraceptive revolution began in earnest.

Mainly for the purpose of safety issues, early progress in contraceptive technology led to dramatic reductions in both the estrogen and progestin components thereby making the pill safer with less side effects. However, two important issues have gradually become dominant issues. First, the estrogen component of the pill is associated with increases in clotting factors and thromboembolic risk. Secondly, high dose progestins by themselves can suppress ovulation. Early appreciation of the increased safety that progestin-only methods of birth control have over combination methods was demonstrated by an essay written in 2005. “The injection is considered safer than the birth control pill because it contains progesterone, whereas the pill contains both progesterone and estrogen.” [3].

While concern for bleeding control had always been an integral part of contraceptive technology, a very strong emphasis on controlling and decreasing menstural bleeding has progressively emerged. Contraceptive technology of today continues to introduce effective products that significantly reduce or eliminated monthly bleeding. As more and more reliance has been placed on the progestin component of the pill to suppress ovulation, substantial reductions have been made in the estrogen dose. The estrogen component of the pill was reduced from 80 μg down to 50 μg, then 35 μg, then 30 μg, then 25 μg, and then 20 μg. In 2010, a 10 μg estrogen combination pill with norethindrone acetate with a 24–4 pill protocol was introduced.

Other attempts were designed to decrease pill hormone content or decrease bleeding episodes. Early changes in the dosing regimen resulted in the introduction of the biphasic and triphasic regimens. The continuous combined protocols were designed to limit bleeding episodes to every 3 or more months. Recent changes include important alterations of the traditional 21–7 monophasic pill regimen. The 24–4 pill regimen shortens the pill free window allowing for a limited withdrawal bleeding episode. By restarting active pills after only 4 days instead of 7 days or placebo or no pills, there is a more limited window when pituitary-stimulation of the ovary is unchecked. The 24–4 pill regimen avoids the large surge in estradiol generally seen toward the end of the 7-day pill free interval. This 24-4 regimen is generally associated with good bleeding control and often less overall bleeding.

In the past, the break-through bleeding that can be seen in women taking depo-MPA or low dose progestin-only pills was attributed to an atrophy of the endometrium due to not enough estrogen that would “heal the endometrium and stabilize bleeding”. The design of many protocols for stopping acute bleeding and managing chronic bleeding problems had an emphasis on early high dose estrogen administration [to build the endometrium] and rely on a progestin to convert the lining. But there now is a change in philosophy.

**Table:** 

***The introduction of progestin only IUDs and the many recent studies addressing the use of high or very high doses of progestins [without added estrogen] that report very effective bleeding control for both acute and chronic problems.*** **Adding more progestin**, not estrogen, to treat abnormal bleeding has become the more reliable protocol. The progestin IUD as well as the menopausal model of no bleeding despite a progestin dominant or atrophic functional endometrial lining adds further evidence to this argument.	

The progestin revolution took off in earnest when the progestin only LARC methods [particularly the hormonal IUDs] offered not only high contraceptive efficacy but also often impressive reductions in overall bleeding. The most recent novel introduction of a high dose progestin only pill is the 4 mg drosperinone oral contraceptive pill that provides a progestin-only option with good bleeding control. This pill also allows a non-time-restricted daily intake, very different from low-dose POP options [4].

## Why suppress menstrual bleeding

**Table:** 

In the 1960s it was suggested that women lacked the ability to hold positions of responsibility and power due to their menstrual cycle. The eminent US endocrinologist Estelle Ramage responded “**In man, the shedding of blood is always associated with injury, disease, or death. Only the female half of humanity is seen to have the magical ability to bleed profusely and still rise phoenix-like each month from the gore”** [5].	

Menstruation has historically been regarded as a sign of inferiority. Many have proclaimed that menses and the change in hormones during the menstrual cycle was linked to incapacitation or physical or intellectual function. A nineteenth century pioneering Scottish gynecologist claimed, “young girls should not play music or read serious books because it makes much mischief with their menstrual cycle”. Fortunately these beliefs have largely been eliminated. However, a new issue has emerged. Women in modern developed countries often choose to have fewer children and thereby experience significantly more menstrual cycles than women in the past. Women in underdeveloped countries often have about 40 menstrual bleeding in a lifetime while many women in developed countries will generally experience more than 400 menstrual bleeds. As modern women now assume positions of responsibility in the workplace and home, abnormal menstruation, menstrual cramps or heavy menstruation can cause significant socio-economic problems [5, 6].

- Menstrual abnormalities are a relatively modern disorder [5]
- Abnormal menstrual bleeding affects 20–30% of premenopausal women), and more than 800,000 women seek treatment annually in the UK [5].
- Abnormal uterine bleeding (AUB) in reproductive-age women (defined as abnormal in duration, quantity, or timing) is experienced by approximately one-third of all women throughout their lifetime that often impair their daily activities.
  - A US study reported financial losses of >$2000 per patient each year due to work absence and home management costs [5].
- The reported prevalence of dysmenorrhea in women is 16–91% while severe pain is reported in 2–29% of women [7].

Although the average period lasts only 4–5 days, normal menstruation can last up to seven days. The average age of menarche is 11–12 years of age but can occur as early as 8 years of age. During the perimenopause, the “roller-coaster” of hormones often results in heavier, more frequent, and less predictable bleeding. Since the average age when bleeding stops is age 50, women often have to deal with 39 years of monthly bleeding episodes. Menses are often associated with cramping and can be associated with nausea or fainting. The average monthly blood loss is 30–40 ml but abnormal bleeding called menorrhagia is by definition > 60 ml. Women with heavy bleeding often use large pads or towels, may need to change pads hourly, and may bleed through clothes or bedding. Menorrhagia can result is severe anemia, need of blood transfusions, emergency surgery, and may in anovulatory women be associated with endometrial hyperplasia or cancer.

**Table:** 

Many new OCPs, hormonal IUDs, depo-MPA, and the new high dose progestin-only pill are useful tools designed to prevent pregnancy and most importantly, to significantly reduce or eliminate bleeding and cramping episodes. Patients often report after treatment “I got my life back.”	

## How to suppress menstrual bleeding

**Table:** 

Menstruation is a physiological model of tightly regulated, unique, and repetitive endometrial shedding as a result of self-limited inflammation activated by progesterone that is followed by rapid healing without scarring [6].	

Understanding the physiological steps of menstruation [rising estradiol, followed by rising progesterone, then falling progesterone] helps to direct effective treatment protocols [5, 6].

1. **Rise in estrogen:** Estrogen stimulation, cell proliferation and development of a thick endometrium
2. **Rise in progesterone**: Progesterone action on the estrogen primed thick endometrium causing decidualization [getting ready for implantation]
  - Progesterone causes endometrial cells to plump with glycogen and lipids.
  - Decidualized cells secrete cytokines, extracellular matrix [promotes ability of embryo to implant]
  - Influx of leukocytes and NK cells making MMPs
3. **Sharp decline of progesterone:** Process to sluff the thick lining
  - The result is tissue breakdown and **shedding of the upper two-thirds of the endometrium** (the functional layer) during the menstrual phase of the cycle.
  - Increased local production and activation of cytokines and MMPs causing focal tissue shedding
    i. Designed to stop the bleeding
      1. Endometrial injury initiates immediate activation and aggregation of platelets to form a plug
  - Platelet glycoprotein interaction with von Willebrand factor
4. **Scarless repair** [6]

Treatment regimens for acute bleeding were originally designed to mimic the hormonal changes of the menstrual cycle by starting treatment with high dose estrogen. However, the high dose estrogen in these protocols has been associated with significant side-effects such as nausea, emesis, and breast tenderness. The high dose progestin is then used to cause the secretory changes and sluffing of the endometrium as discussed above. But importantly, making more endometrium does not appear to be needed. The high dose estrogen likely stimulates a cell proliferation and may increase the amount of the progestin needed to convert the functional layer and thus use of high estrogen may eventually result in more bleeding compared to treatment with just progestin alone. Many progestin only protocols have been shown to be at least as effective in stopping acute bleeding as protocols using high dose estrogen and progestin combinations, and importantly are often associated with fewer side effects. Progestin only regimens along with the combination protocols are included as first line treatment options as listed below. More recent studies [discussed under New and Effective MPA Protocols below] report on new progestin-only protocols that are effective and well tolerated.

- ACOG Committee Opinion recommends multiple therapies to manage acute AUB [8] in adults and reaffirmed for adolescents [9]
  - IV conjugated equine estrogen [25 mg every 4–6 h × 24 h]
  - Monophasic OCPs with 30–50 μg ethinyl estradiol [every 6–8 h until cessation of bleeding]
  - Tranexamic acid [1.3 g oral or 10 mg/kg IV 3x daily for 5 days]
  - **Medroxyprogesterone acetate [20 mg 3 x/day × 7 days]**
- A recent European Consensus group offered 4 oral options for hormonal treatment of acute bleeding in women without underlying bleeding disorders [10] Each treatment option had a recommended taper protocol.
  - Birth control pills with either 30 mcg or 50 mcg of ethinyl estradiol (EE) in combination with any progestin to be taken every 6 h until bleeding stops (with a re-evaluation at 48 h)
  - **Norethindrone acetate 5 mg–10 mg every 4 h**
  - **MPA 10 mg every 4 h (up to 80 mg per day)**

## Low-dose progestin-only pills often have poor bleeding control

The history of progestin-only pills began in 1973 when a low dose progestin-only “mini-pill” with 0.35 mg norethindrone was introduced. Table 1 As opposed to oral combination pills, there is an extra requirement of this pill as it has to be taken within a strict three-hour daily window. If the window is missed, it counts as a missed pill and increases the risk for method failure. The pill has variable contraceptive protection [linked heavily to compliance and this daily window] and is often associated with irregular bleeding episodes. The contraceptive efficacy is linked to the very low dose of daily norethindrone and its relatively short 5–13 h ½ life. This low dose progestin-only pill does not suppress pituitary activity to the same extent as combined pills and it depends heavily on a progestin effect on cervical mucus that is lost when progestin levels become too low. Table 1.

**Table 1:** Low dose progestin-only oral contraceptive

**Mechanisms of conceptive protection**	
Suppresses ovulation [in approximately half of users],	
Thickening the cervical mucus to inhibit sperm penetration	
Lowering the midcycle LH and FSH peaks	
Slowing the movement of the ovum through the fallopian tubes	
Altering the endometrium	
**Action of norethindrone**	
Peak levels of norethindrone occur 24 h after oral dose followed by a rapid distribution and elimination. Efficacy is dependent upon compliance of an every 22–24 h dosage regiment	

Although many “mini-pills” have been introduced around the world, most have been withdrawn due to problems with compliance [need to be taken at same time each day], failure rates, irregular bleeding patterns, or complaints of mood disturbances. Table 2 It wasn’t until May, 2019 that a high progestin only o**ral contraceptive** was approved by the FDA. This novel high dose progestin only pill (POP) contains 4 mg drospirenone in each active tablet [that is a higher dose than in the currently marketed drospirenone-containing combined oral contraceptive pill [3 mg drospirenone]. Another big advantage of this high dose progestin pill is that it is taken in the 24–4 daily protocol. The 4 pill free days are designed allow a controlled withdrawal bleeding episode [compared to the 28-day minipill regimen with no window for bleeding]. This high dose drosperinone oral contraceptive pill provides a progestin-only option with good bleeding control without the restricted daily intake 2–3 h time frame [4].

**Table 2:** Progestin only Pills

**Progestin only pills now commercially available**	
**Minipills**	
Norethindrone 350 μg	
Desogestrel [not in USA] 75 μg	
**High progestin-only pill**	
Drosperinone 4 mg	
**Progestin only pills mostly discontinued [none available in USA]**	
**Minipills**	
Etynodiol diacetate 500 μg	
Levonorgestrel 30 μg	
Lynestrenol 500 μg	
Norethisterone 300 μg	
Norgestrel 75 μg or levonorgestrel 37.5 μg	
**Discontinued Minipills**	
Chlormadione acetate 0.5 mg	
Quingestanol acetate 0.3 mg	

### Developing dominance of [high dose] progestin-only methods in suppressing menstrual bleeding

#### Advantages of Medroxyprogesterone acetate [MPA] Table 3

Progestins are increasingly becoming the treatments of choice to stop acute bleeding and/or control long-term bleeding. Progestin-containing IUDs are very effective for long-term bleeding control and are discussed at length below. While Depo-MPA can be associated with irregular bleeding, amenorrhea is an advantage to many users as 50% of users are amenorrheic at 6 year of use and 70% are amenorrheic after 2 years of use [14]. Depo-MPA decreases dysmenorrhea, anemia and the risk of endometrial and ovarian cancer [15, 16].

**Table 3:** Depo-Medroxyprogesterone Acetate and Medroxyprogesterone Acetate Facts

Benefits / Advantages/History	Risks
DMPA was discovered in 1956 and approved in the USA in 1960 for treatment of endometrial and renal cancer and in 1992 as a contraceptive. An oral tablet MPA 2.5,5 and 10 mg medroxyprogesterone acetate was approved in the US in 1959 for the treatment of metorrhagia, amenorrhea, and recurrent miscarriage. A combination OCP was introduced in 1964 in the U.S. [brand name Provest] (10 mg MPA and 50 μg ethinylestradiol tablets) but it was discontinued in 1970. In 2017, it was the 222nd most commonly prescribed medication in the United States, accounting for more than two million prescriptions. It is currently approved in more than 100 countries around the world and often listed among the most commonly prescribed medications [11].	Reports of serious thrombotic events in women using Depo-Provera, but not causally associated with the induction of thrombotic or thromboembolic disorders [12]. The package insert for the contraceptive Depo-MPA states: “The physician should be alert to the earliest manifestations of thrombotic disorder (thrombophlebitis, cerebrovascular disorder, pulmonary embolism, and retinal thrombosis). Should any of these occur or be suspected, the drug should be discontinued immediately.”
The WHO has recommended that the use of DMPA not be restricted. It is on World Health Organization’ s List of Essential Medicines that selects the safest and most effective medicines needed in for healthcare [13].	Bone loss after 2 years of use and weight gain are reported side effects and should be part of a risk benefit decision
Associated with a reduced frequency of seizures; does not interfere with antiepileptic mediations.	Mood changes are reported in some women on progestins including both oral and injectable MPA.
December 2004, a subcutaneous version of DMPA was approved in the USA as a contraceptive (104 mg/0.65 mL MPA); and also approved for the treatment of endometriosis-related pelvic pain.	Irregular bleeding, particularly during the first months of use, in common and can continue with prolonged treatment.
Improves blood counts in women with sickle cell anemia; used to improve iron deficiency anemia due to menstrual blood loss	
Associated with a decreased risk of pelvic inflammatory disease	

Norethindrone acetate [NET acetate] [brand name Aygestin] and MPA are used effectively to stop acute bleeding and recommended protocols are discussed above. However, MPA, in both the injectable and oral form, has an important advantage over NET Acetate for management of acute and chronic bleeding control.

**Table:** 

**Fig. 1:**
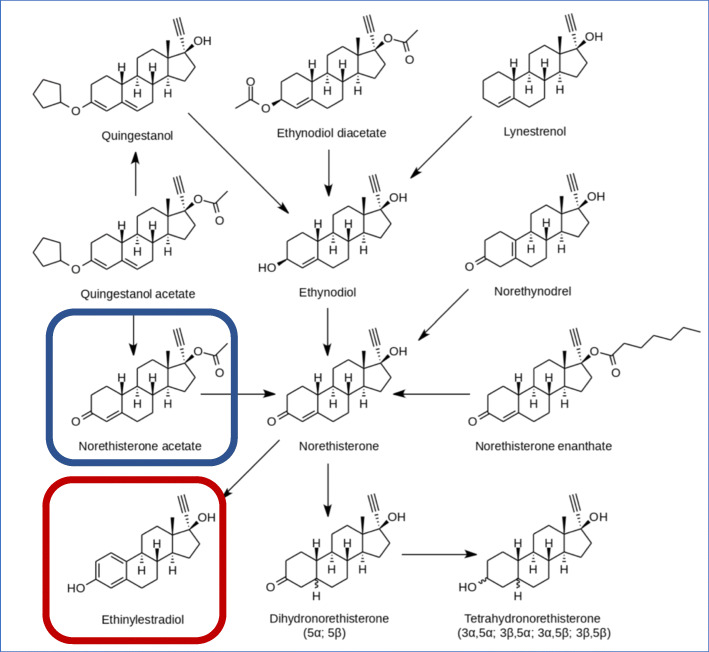
Conversion of multiple progestins, particularly NET acetate, to ethinyl estradiol 11–13

**The advantage is that MPA acetate is** **not metabolized to ethinyl estradiol** **as are many other progestins including NET acetate** [also known as Aygestin and norethisterone acetate]**.** Figure 1 [17, 18].	

**Fig. 2:**
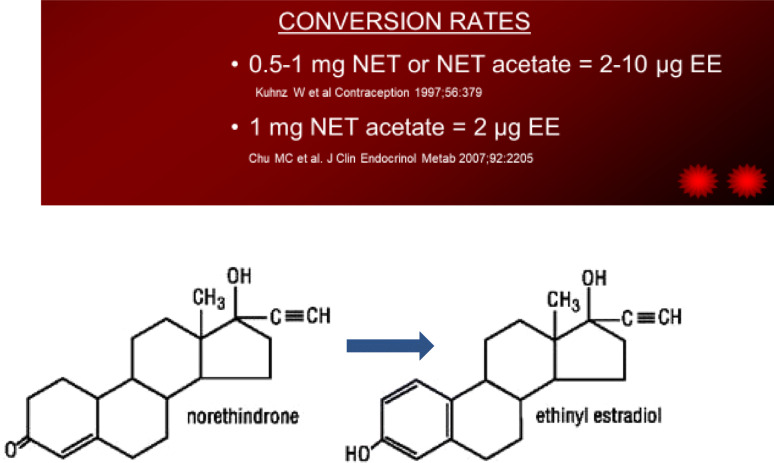
Conversion rates of norethindrone and norethindrone acetate to ethinyl estradiol via aromatase 14–15

Figures 1 and 2 show the conversion of NET or NET acetate to ethinyl estradiol in postmenopausal women [17] and premenopausal women [18]. The use of NET acetate 5 mg tablet gives rise to about 10 μg of ethinyl estradiol. Table 4 Thus the use of NA 5 mg/day results in hormonal levels similar to a very low dose OCP. While NET acetate 5 mg daily has not been shown to have contraceptive action and should not be used in that way, NET acetate can be used as a hormone replacement product in younger women with premature menopause or oophorectomy when estrogen is needed to protect bones and other estrogen sensitive tissue [19].

**Table 4:** NET acetate [Aygestin] Facts

NET acetate is supplied in 2.5 and 5 mg. NET acetate produces secretory changes in an estrogen-primed endometrium. It is twice as potent as norethindrone on a weight basis. It is used for the treatment of secondary amenorrhea, endometriosis, and abnormal uterine bleeding due to hormonal imbalance in the absence of organic pathology.	
NET acetate may be given daily for 5 to 10 days to produce secretory transformation of an endometrium. Withdrawal bleeding usually occurs within three to seven days after discontinuing. Patients with chronic abnormal uterine bleeding can be treated with a daily dose of 2.5 to 15 mg/day of NET acetate can be continued for 6 to 9 months. Some breakthrough bleeding generally occurs.	

#### MPA: new effective MPA protocols for abnormal bleeding control

For immediate and long-term control of bleeding in women, many new publications report the effectiveness of protocols using MPA acetate with the injection-only [long-term control], high dose oral MPA only [short term taper protocol] or combination of both MPA injection and oral pills [for hemodynamically stable women with acute heavy bleeding, anemia, or “when you really need to stop the bleeding”].

As discussed above, the advantage of using MPA acetate over NET acetate is that MPA acetate promotes a strong progestational effect without being converted in part to estrogen. Fig. 2 After a 5 mg NET acetate pill intake, studies show that there is formation of around 10 μg ethinyl estradiol. This rise in a very potent estrogen can cause stimulation and growth of the functional endometrium and likely increases the amount of progestin needed to completely transform the functional layer. Inadequate progestin action on the endometrium can result in continued bleeding. Importantly, studies show that priming with estrogen is not necessary. Progestin alone is effective in stopping and controlling bleeding as shown in the following studies.

Studies showing MPA acetate alone effectiveness.

1. Contrary to conventional wisdom, women treated only with MPA acetate responded at least as well as women in the COC arm and had less side effects [20].
  Prospective, randomized, comparative study of hormonal options, high-dose COCs (*n* = 20) were compared to high-dose MPA (*n* = 20).
  Hemodynamically stable patients with acute uterine bleeding sufficient to justify immediate medical or surgical intervention were enrolled
  ○ Oral medroxyprogesterone acetate 20 mg 3 times x/day
  ○ or a monophasic combination OC containing 1 mg norethindrone and 35 μg of ethinyl estradiol 3 times/ day.
  Doses were reduced after 1 week to 20 mg/day MPA or one OCP tablet per day for the next 3 weeks.
  All women in the MPA group avoided surgery. In the COC group, 5% (1 woman) needed an emergency surgical procedure.
  **The percentage of women who stopped bleeding was the same in MPA and COC groups (75% vs 88%, RR 0.87 [95% CI, 0.56–1.31]).**
  Median time to bleeding cessation was 3 days in both groups.
  Where the groups did differ, however, was in patient satisfaction; 81% of the MPA patients said they would use the medication again, while only 69% of the COC users said they would do so (RR 1.18 [95% CI, 0.73–0.98]).
2. Rapid saturation of the endometrium with high dose progestogens seems to be a highly effective mode of treatment for excessive dysfunctional uterine bleeding in adolescents [21] High-dose medroxyprogesterone acetate for the treatment of dysfunctional uterine bleeding in 24 adolescents [21].

- All of the teen women hospitalized for acute uterine bleeding stopped bleeding within 4 days when given a high dose MPA initial dose
  - ○ 60 mg to 120 mg MPA on day 1, followed by
  - ○ 20 mg MPA daily for an additional 9 days.

3. An effective progestin-only option for hemodynamically stable women with very heavy, acute bleeding and anemia [22].

- 48 women, 19–53 years, non-randomized study
- Mean BMI of 34.9 kg/m^2^ (range 21.5–51.2 kg/m^2^).
- Baseline hemoglobin = 10.9 g/100 mL
- 2 pills 3 times daily × 3 days 10 mg oral MPA
- Plus IM 150 mg Depo-MPA
- The mean duration of bleeding was 30.6 days **All 48 women stopped bleeding within 5 days**
- **Mean time to bleeding cessation was 2.6 days.**
- **Side effects were infrequent and patient satisfaction was high**.
- Injection of depo-MPA 150 mg intramuscularly combined with 3 days of oral MPA acetate 20 mg every 8 h for 9 doses is an effective outpatient therapy for acute abnormal uterine bleeding.

## Development of LARC methods: the other side of the progestin revolution

### A breakthrough study started the development of the LARCs: release of steroids from a silicone polymer

The LARC side of the progestin revolution had a lucky beginning. In 1972, a paper was published in the Journal of Pharmaceutical Science describing the In vitro release of four progesterone-type steroids from a silicone polymer. Luckily, the molecular size of each of these steroids was able to penetrate through the 3-D structure of the silicone polymer, each at their own steady state rate. [23].

*“The amount of drug released from this matrix system was found to be dependent upon the molecular structure of the steroid. Progesterone, for example, was released approximately eight times faster than 17α-hydroxyprogesterone under identical experimental conditions. Since the diffusion coefficients of the steroids were of the same magnitude, the diversity in release patterns was mainly attributed to the differences in the polymer solubilities of the steroid.”* [23]

The pairing of progestins and plastic polymers was first realized in 1976 with the introduction of Progestasert. The 12-month Progestasert IUD was made of the ethylene/vinyl coacetate polymer (EVA) [also in Nexplanon] with a reservoir of 38 mg of microcrystallized progesterone. Appreciation of bleeding control was appreciated even then.

*Progesterone may decrease the endometrial content of prostaglandins and decrease the concentration of blood vessels in the endometrium which may result in fewer complaints of dysmenorrhea and a lower total volume of menstrual blood loss in women using a progesterone IUD* [24].

## The progestin revolution peaks with introduction of a succession of LARC progestin-only contraceptive methods with excellent bleeding control

### Progestin-only LARC time-line

#### Mirena 2000

The Mirena intrauterine system releasing levonorgestrel into the uterus was approved in 2000 as a 5-year contraceptive method. It was one of the most effective methods with a failure rate around 0.2% This IUD had been approved in Finland in 1990. Mirena consists of a T-shaped polyethylene frame (T-body) with a steroid reservoir containing a mixture of 52 mg levonorgestrel and silicone. **2009 - U.S. FDA approved Mirena IUD to treat heavy periods (menorrhagia).** 2020 – FDA approved Mirena for 6 years of use.

#### Implanon 2006

Implanon contraceptive implant releasing etonogestrel approved for 3 years 2011 – Nexplanon contraceptive implant replaced the Implanon.

#### Skyla 2013

Skyla contains 19.5 mg levonorgestrel that is released for up to 3 years of use.

#### Liletta 2015

Liletta levonorgestrel intrauterine device (IUD) with 52 mg, similar in size to the Mirena IUD, was approved by the U.S. FDA 2015 for 3 years of use. In 2017, it received approval for 4 years use, in 2018 approved for 5 years use, and in 2019 approved for 6 years. Bleeding data for Liletta indicate that amenorrhea rates are about 40%, even into year six.

#### Kyleena 2016

FDA approved Kyleena IUD containing 19.5 mg levonorgestrel - slightly smaller than Mirena but still with 5 years contraceptive approval.

Multiple studies have shown high efficacy of hormonal IUDs in suppressing bleeding in both women with normal bleeding and in those with heavy menstrual bleeding.

- A Cochrane review compared the efficacy of multiple combined hormonal contraceptives compared with other therapies in the treatment of heavy menstrual bleeding (HMB). While combined oral hormonal contraceptives were effective in reducing HMB, the levonorgestrel (LNG) releasing IUD was more effective [24]. Their findings are below.

**Table:** 

The levonorgestrel-releasing intrauterine system (LNG IUS) reduced HMB more effectively than the combined oral contraceptive pill (COCP) [25].	

Combined oral hormonal contraceptives reduce HMB to ‘normal’ in 12 to 77% of women (when compared to 3% of women taking placebo).

Limited evidence suggested that the combined hormonal vaginal ring (CVR) was as effective as COCPs.

Short-term combined hormonal contraceptives (either COCP or CVR) can effectively reduce HMB, although not as much as the LNG IUS. Both treatments are useful for women who want to reduce their HMB, prevent pregnancy, and preserve future fertility.

There are other non-hormonal medical treatments that also offer moderate efficacy and these could be considered for women for whom oestrogen and progestogen are contraindicated. Moreover, for women towards the end of their reproductive lives, minimally-invasive surgical treatment may be preferable. Choice of treatment for HMB should ultimately be based on women’s preferences, other comorbidities, need for contraception, and pattern of symptoms.

- In women suffering from heavy bleeding, Mirena reduced the amount of bleeding by 80% after 3 months of use. After 6 months, bleeding was reduced by 90% [26].
- 50 women planning on having surgery to treat their heavy periods agree instead to have Mirena inserted instead. Thirty-seven of the women reported that they noticed much lower amounts of blood loss after 3 months of Mirena use. This number increased to 41 after 9 months of use. Forty-one of these women decided to continue using Mirena instead of having surgery to treat their heavy bleeding [27].

- Six different research studies showed that when compared with endometrial ablation, Mirena was as effective in reducing monthly blood loss. Mirena was associated with fewer side effects and it did not affect future fertility [28, 29].

- In a 1-year study in women with heavy bleeding, Mirena decreased blood loss in three out of four women —79.5% of the women planned to continue using Mirena. Hemoglobin levels increased at 3 and 12 months in women with the Mirena [30].

- Mirena, hysterectomy, and endometrial ablation for heavy bleeding were compared. Mirena was ranked as best regarding the number of quality-of-life years, next was hysterectomy, followed by endometrial ablation [31].

## Conclusion

The tight link between contraception and bleeding control has led to the development of safe and effective contraceptive options that often offer excellent bleeding control. The progestin only LARC methods were particularly valuable in demonstrating bleeding control with the use of high dose progestin action on the endometrium. Progestin-only protocols, particularly those using MPA oral pills with or without injectable MPA, are very effective in stopping acute and controlling long-term bleeding and are associated with less side effects than many of the traditional protocols.
